# Dietary Supplementation With Fish Oil Enhances the Growth and Reproductive Performance of Female Broodstock *Haliotis discus hannai*

**DOI:** 10.1155/anu/9987051

**Published:** 2025-07-15

**Authors:** Weiguang Zou, Jiacheng Gan, Yanbo Liu, Yawei Shen, Jiawei Hong, Yaobin Ma, Chunxiang Ai, Xuan Luo, Weiwei You, Caihuan Ke

**Affiliations:** ^1^State Key Laboratory of Mariculture Breeding, College of Ocean and Earth Sciences, Xiamen University, Xiamen, China; ^2^Hainan Academy of Ocean and Fisheries Sciences, Haikou 570100, China

**Keywords:** abalone, growth performance, lipid sources, mollusk, ovarian development, reproductive performance

## Abstract

Macroalgae have long been utilized as a natural feed source in abalone aquaculture. The switch to formulated feeds improves nutritional control while reducing the cost instability of natural feeds. Recently, lipid supplementation has received a lot of attention since it is essential to the early developmental and broodstock stages of aquatic species. The present study aimed to investigate how the growth and reproductive performances of female broodstock *Haliotis discus hannai* were affected by the dietary supplementation of five lipids: fish oil (FO), perilla seed oil (PO), safflower oil (SO), olive oil (OO), and lard (LO). A total of 500 adult female abalones were randomly divided into five groups (four replicates per group) and fed with five lipid-supplemented diets for 90 days. The LO group was the worst (*p* < 0.05), while the FO group significantly showed the best growth performance, gonad index (GI), fertility, fertilization rate, larvae hatching rate, and larval attachment rate (*p* < 0.05). Meanwhile, the FO and PO groups exhibited significantly higher levels of very low-density lipoprotein (VLDL), vitellogenin (VTG), gonadotropin-releasing hormone (GnRH), progesterone (PROG), and 17β-estradiol (E_2_) than the LO group (*p* < 0.05), indicating their higher ovarian developmental maturity. Furthermore, histological and fatty acid analysis revealed that the FO group contained high levels of polyunsaturated fatty acids such as eicosapentaenoic acid (EPA) and docosahexaenoic acid (DHA), which may contribute to ovarian maturation and egg quality. Therefore, the best lipids for improving the growth and reproductive performances of female abalone might be FO enriched in highly unsaturated fatty acids rather than plant-derived lipids, providing new insights into nutritional research and applications in vegetarian mollusks.

## 1. Introduction

In recent years, the global abalone industry has experienced a marked shift from fisheries to aquaculture-based production [1]. Abalone, particularly in China, has become a cornerstone species in commercial aquaculture, with over 95% of global abalone production coming from aquaculture, of which China accounts for 88% [2]. However, this rapid expansion of abalone aquaculture has brought about several challenges, including slow growth rates, suboptimal egg quality, and reduced larval survival. Addressing these issues requires meeting the nutritional requirements of broodstock abalone to support larval development and improve survival [3]. Traditionally, macroalgae have been used as a cost-effective and natural feed source in abalone aquaculture. However, reliance on natural macroalgae presents challenges, such as decreasing availability and inconsistent nutrient composition. In contrast, formulated feeds offer several advantages, including controlled and consistent nutritional profiles, along with greater convenience in terms of purchasing, handling, and storage [4]. Thus, the shift to formulated feeds not only mitigates the limitations of natural feed sources but also enhances nutritional management, which is crucial for optimizing the healthy growth and reproductive performance of abalone. Given the variability in nutrient requirements across different developmental and physiological stages of aquatic animals [5], it is essential to meet the dynamic nutritional needs of female broodstock abalone.

Extensive research on dietary lipid sources has demonstrated variations in essential fatty acid (EFA) requirements across different species and stages [5]. Lipids play a critical role in reproductive performance and larval survival in marine species, particularly during the early developmental and broodstock stages [6–8]. The fatty acid composition in dietary lipids significantly influences fecundity, fertilization rates, reproductive performance, and larval survival [9]. EFAs are indispensable for the reproductive health of broodstock abalone and are essential for ensuring larval viability, as they play a key role in maintaining cell membrane integrity and supporting larval development [3]. Additionally, long-chain polyunsaturated fatty acids (LC-PUFAs), such as arachidonic acid (ARA, 20:4*n −* 6), eicosapentaenoic acid (EPA, 20:5*n −* 3), and docosahexaenoic acid (DHA, 22:6*n −* 3), are particularly crucial for the structural integrity of egg cell membranes, organogenesis, and the synthesis of eicosanoid hormones of abalone [10].

Fish oil (FO), a traditional and widely used lipid source in aquaculture, is rich in *n −* 3 long-chain PUFAs such as EPA and DHA, providing not only energy but also enhancing the palatability of diets [11]. Studies have investigated the potential of replacing FO in *Nibea albiflora* feed with alternative oils such as soybean, linseed, and sunflower oil, which provide cost-effective and stable sources of C18 PUFAs [12]. However, the lack of specific EFAs in vegetable oils may negatively impact the reproductive performance of aquatic species [13, 14]. Research indicates that dietary linolenic acid (LNA) and linoleic acid (LA) can influence steroid hormone synthesis during the ovarian development of yellow catfish *Pelteobagrus fulvidraco* [15]. Earlier studies have suggested that abalone may have the capacity to convert LA to ARA or alpha-linolenic acid (ALA) to EPA [16]. Additionally, higher *n* − 3 to *n −* 6 PUFA ratios have been associated with improved egg quality and increased offspring survival in various aquatic species, including *Oncorhynchus mykiss* [13], *Trachinotus ovatus* [17], and *Cyprinus carpio* [18].

Despite these advancements, studies on the dietary lipid sources of abalone have focused mainly on the growth performance of juveniles [19], while there is a paucity of research on the growth and reproductive capacity of female broodstock abalone. Therefore, in this study, FO (enriched with LC-PUFA), perilla seed oil (PO, enriched with LNA), safflower oil (SO, enriched with LA), olive oil (OO, enriched with oleic acid), and lard (LO, enriched with SFA) were selected as different lipid sources to investigate their effects on growth performance, biochemical composition, reproductive performance, fecundity, histological characteristics, and hormone levels in female broodstock *H. discus hannai*. The objective is to clarify the role of dietary lipids in improving reproductive efficiency, and to provide new insights into the optimal lipid sources for the dietary management of female broodstock abalone.

## 2. Material and Methods

Animals involved throughout the experimental procedures and protocols were endorsed by the Institutional Animal Care Committee of Xiamen University under the guidance of the care and use of China laboratory animals (XMULAC20240335).

### 2.1. Experimental Diet Preparation and Analysis

Five isonitrogenous (36% crude protein) and isolipidic (4% total lipid) experimental diets were formulated with different lipid sources as follows: FO, PO, SO, OO, and LO. The ingredients and proximate compositions of these diets were shown in Table 1, while the fatty acid profiles were presented in Table 2. The diet was prepared in accordance with the method used by Zou et al. [20]. Briefly, all components were finely ground, sieved (100 μm), and mixed according to Table 1 followed the principle of mixing from small to large and gradually expanding is to mix evenly. After mixing, the mixture was transferred to a high-shear mixer. The required amounts of oil and water were weighed and then emulsified using a high-shear emulsifier (BRS-500, 15,000 rpm) to form an oil–water mixture. While stirring, the mixture was then pressed into flakes (4 cm × 4 cm × 0.5 cm) using a tablet press. The flakes were dried at 50°C for 24 h, and subsequently stored at −20°C until use.

The analyses for moisture, ash, and total lipid were performed using standard methods [21]. Moisture content was measured by oven drying (105°C), and ash content was determined by incinerating samples at 550°C for 6 h. Total lipid content was extracted with ether following Sotheby's method. Crude protein was assessed via high-temperature combustion and infrared thermal conductivity analysis, with nitrogen content converted to crude protein using a factor of 6.25. The method might have overestimated the true protein content, but the nonprotein nitrogen content of the dietary ingredients in this study was low, and these deviations were within acceptable limits.

Retention efficiency (RE) of the diets ranged from 85.21% to 85.49% at 24 h and 82.16% to 82.60% at 48 h (Table S1), with no significant differences in RE among diets (*p* > 0.05), indicating that the five diets in this study were prepared effectively.

### 2.2. Experimental Abalone and Feeding Trial

In this study, 1.5-year-old abalone were obtained from Fuda Aquaculture Company (Fujian, China). Prior to the feeding experiment, the abalone were temporarily reared in concrete tanks for 15 days to acclimatize to the compound feed and culture environment according to the method described by Zou et al. [20] and Zou et al. [22]. A total of 500 female broodstock abalones, with an initial shell length of 60.33 ± 0.22 mm (mean ± standard error[SE]) and an initial body weight of 28.17 ± 0.29 g were selected. Abalones were randomly distributed into black plastic cages (length × width × height = 70 cm × 35 cm × 45 cm), with 25 individuals per cage and four replicates per group. These cages containing abalone were then placed in a concrete pond (8 m × 2.2 m × 0.8 m) for culture. During the 90-days feeding trial, the abalones were fed experimental diets at 1%–3% of their total body weight once a day at 18:00. To ensure apparent satiation, the amount of feed provided was adjusted based on the remaining feed from the previous feeding. To assess feed intake, residual feed was collected, dried, and weighed before feeding. To maintain optimal conditions, two-thirds of the seawater was replaced daily, and water parameters, including temperature (21.5–22.5°C), dissolved oxygen (7.34–7.39 mg/L), salinity (31–32‰), and pH (7.6–8.0), were carefully monitored and controlled.

### 2.3. Sample Collection

At the end of the 90-days feeding experiment, all abalones were starved for 72 h before being counted, measured, and weighed to determine final shell length (FSL), final body weight (FW), survival, daily increment in shell length (DISL), weight gain rate (WGR), specific growth rate (SGR), and feed conversion rate (FCR). Twelve abalones (three abalones per replicate) were randomly selected from each experimental treatment group, anesthetized on ice, and hemolymph samples were collected with a sterilized syringe. The samples were then immediately centrifuged at 3000 *g* for 10 min to collect serum for biochemical analysis. Afterward, the livers and ovaries of the abalones were collected from the hemolymph-taken abalones, immediately frozen in liquid nitrogen, and stored at −80°C for further analysis. The formulas for growth performance calculation were as follows:

  $$\begin{array}{c} \text{Survival }\left(\text{\%}\right)=\frac{\text{Number of abalone at the end}}{\text{Initial number of abalone}}\times 100\text{\% }, \end{array}$$

  $$\begin{array}{c} \text{DISL }\left(µ\text{m}/\text{day}\right)=\frac{\text{Final shell length}-\text{initial shell length}}{\text{90 days}}\times 1000, \end{array}$$

  $$\begin{array}{c} \text{WGR }\left(\text{\%}\right)=\frac{\text{Final body weight}-\text{initial body weight}}{\text{Initial body weight}}\times 100\text{\% }, \end{array}$$

  $$\begin{array}{c} \text{SGR}\;\left(\text{\%}\right)=\frac{\ln\left(\text{final weight}\right)-\ln\left(\text{initial weight}\right)}{\text{90 days}}\times 100\text{\% }, \end{array}$$

  $$\begin{array}{c} \text{FCR}=\frac{\text{Weight of feed dry matter ingested}*}{\text{Final body weight}-\text{initial body weight}}. \end{array}$$

*⁣* ^*∗*^The weight of feed dry matter ingested was the difference between the total weight of feed fed (dry matter) minus the total weight of feed remaining (dry matter) and minus the 24 h feed loss rate.

### 2.4. Hepatosomatic Index (HSI), Gonad Index (GI), and Histological Analysis

The HSI was calculated as the ratio of hepatopancreas weight to total body weight. For histological analysis, the apical portions of the hepatopancreas, including the liver and gonads, were excised and fixed in Bouin's solution for 24 h. The tissues then underwent dehydration, clarification, and paraffin embedding. Transverse sections (5 μm thick) were prepared using a microtome and stained with hematoxylin and eosin for microscopic examination. Additionally, the remaining hepatopancreas tissue was preserved in alcohol for GI calculation. The formulas were calculated as follows:

  $$\begin{array}{c} \text{Hepatosomatic index}\;\left(\text{\%}\right)=\frac{\text{Weight of hepatosomatic}}{\text{The weight of whole body}}\times 100\text{\%}, \end{array}$$

  $$\begin{array}{c} \text{GI }\left(\text{\%}\right)=\frac{\text{Transverse area of ovary}}{\text{Transverse area of whole hepatopancreas}}\times 100\text{\%}. \end{array}$$

### 2.5. Determination of Reproductive Performance

Reproductive performance was assessed following the method of Zou et al. [23]. Briefly, 20 female abalones were randomly selected from each treatment group five abalones per replicate, four replicates total), along with six male abalones from the FO treatment group (to ensure sperm quality consistency) reared under identical conditions with well-developed gonads. All broodstock abalones underwent 1.5 h air exposure in a cold, dry environment before being transferred to 20 L plastic tanks. Female broodstock abalones were distributed across four tanks (five abalones per tank, four replicates per treatment), while male broodstock abalones were housed separately in one tank. UV-irradiated seawater was introduced to induce spawning and ejaculation. When sperm density reached optimal concentration, semen samples were collected using disposable pipettes and quantified via hemocytometer under optical microscopy. Sperm suspensions were standardized to 7.0 × 10^5^ individuals/ml through concentration adjustment.

#### 2.5.1. Determination of Fertilization Rate, Hatching Rate, Abnormality Rate, and Attachment Rate

Within 1 h after spawning, eggs were collected, and approximately 5.000 eggs were collected from each plastic tank into a beaker containing 20 mL of filtered seawater (one beaker per replicate, four replicates per treatment group). Subsequently, 200 μL of 7.0 × 10^5^ individuals/mL of sperms were added to each beaker, and the contents were gently mixed. To remove excess sperm, fertilized eggs were washed three times with filtered seawater at 25-min intervals. Two hours after fertilization, a 1 mL sample was taken from each beaker, and fertilized eggs that had completed or failed to complete the first cell division were counted to calculate the fertilization rate. The fertilization rate was calculated using the following formula:

  $$\begin{array}{c} \text{Fertilizationrate }\left(\text{\%}\right)=\frac{\text{Number of divided eggs}}{\text{Total number of eggs}}\times 100\text{\%}. \end{array}$$

Twelve hours after fertilization, 1 mL samples of seawater were drawn from each beaker to assess the hatching and the abnormality rates. The rates were calculated using the following formulas:

  $$\begin{array}{c} \text{Hatching rate }\left(\text{\%}\right)=\frac{\text{Number of hatched larvae}}{\text{Number of divided eggs}}\times 100\text{\%}, \end{array}$$

  $$\begin{array}{c} \text{Abnormality rate }\left(\text{\%}\right)=\frac{\text{Number of abnormal larvae}}{\text{Number of hatched larvae}}\times 100\text{\%}. \end{array}$$

Twenty-four hours after fertilization, 100 planktonic larvae were collected from each beaker and transferred into a PVC pipe sealed at the bottom with 300-mesh bolting silk. Once the water was free of larvae, aeration was initiated and maintained for 15 days, with the temperature held at 20–21°C, dissolved oxygen at 7.15–7.28 mg/L, and salinity at 31–32 g/L. After 15 days, the attachment rate was calculated using the following formula:

  $$\begin{array}{c} \text{Attachment rate }\left(\text{\%}\right)=\frac{\text{Number of attached larvae}}{\text{Total number of larvae}}\times 100\text{\%}. \end{array}$$

#### 2.5.2. Determination of Total Fecundity and Relative Fecundity

Twenty female broodstock abalones were randomly selected from each experimental treatment group for spawning. Eggs were collected using a 300-mesh sieve and transferred into a 5L plastic container. To induce further spawning, UV-treated water was added to the container, with the process repeated every 2 h until all eggs were collected. The eggs were then placed in a PVC pipe sealed at the bottom with 300-mesh bolting silk. This pipe was positioned in a plastic basin containing 2 L of fresh seawater, with an internal volume of 443.72 cm³. After thorough mixing, a 1 mL sample of the egg suspension was taken for counting. The formulas used were as follows:

  $$\begin{array}{c} \text{Total fecundity}=\frac{\text{Total number of eggs}}{\text{Total number of female broodstock abalones}}, \end{array}$$

  $$\begin{array}{c} \text{Relative fecundity}=\frac{\text{Total number of eggs}}{\text{Total weight of female broodstock abalones}}. \end{array}$$

### 2.6. Determination of the Biochemical and Hormone Parameters

Liver and ovary tissues (about 0.1 g) were homogenized in saline (0.86 v/w) and then centrifuged to obtain the supernatant. Triglyceride (TG) levels in both serum and liver were measured using commercial assay kits (A110-1-1, Nanjing Jiancheng Bioengineering Institute, Nanjing, China). Additionally, very low-density lipoprotein (VLDL), 17β-estradiol (*E*_2_), and progesterone (PROG) in serum and liver, as well as gonadotropin-releasing hormone (GnRH) in serum and PROG, *E*_2_, and vitellogenin (VTG) in the ovary, were measured using commercial kits (Shanghai C-reagent Biotechnology, Shanghai, China). The kit codes were TG (A110-1-1), VLDL (CS-00C991110), *E*_2_ (CS-SM9563), PROG (CS-00S996710), GnRH (CS-SM9561), and VTG (CS-00C991113). All procedures followed the manufacturer's instructions.

### 2.7. Analysis of Fatty Acid Composition

Fatty acid analysis was performed using a method adapted from Liu et al. [7], with slight modifications. Briefly, 50 mg of sample powder was added to a 10 mL volumetric flask containing 1 mL of ethanol and 5 mL of hydrochloric acid (8.3 mol/L), then heated in a water bath at 75–80°C for 40 min. After cooling, 10 mL of ether-petroleum, ether (1:1) was added and shaken for 5 min, followed by the collection of the supernatant. Saponification was performed with 5 mL of sodium hydroxide-methanol solution (2%) at 80°C for 1 h, then 3 mL of boron trifluoride-methanol solution (14%) was added and heated for 10 min. The mixture was shaken with 2 mL of n-heptane and 3 mL of saturated sodium chloride solution to separate the layers. Fatty acid methyl esters (FAMEs) were filtered using a 1 mL syringe with a 0.2 µm filter membrane. FAMEs were quantified using a gas chromatograph (Agilent G7000D, USA) with a CP-Wax 52 CB column (30 m, 0.25 μm thickness and 0.25 mm inner diameter). The temperature program began at 50°C (3-min hold), increased to 170°C for 12 min, 170 – 205°C for 11.67 min, 205 – 235°C for 6 min, and finally 235 – 250°C for 1.5 min. The fatty acid composition of the samples was determined according to the retention time of 37 fatty acid standards (Product No. 18919-1AMP, Supelco, USA) and the relative fatty acid content was calculated using the area percentage method.

### 2.8. Statistical Analysis

Data analyses were performed using SPSS 20.0 (SPSS, Chicago, IL, USA), and values were reported as means ± SE. One-way analysis of variance (ANOVA) was conducted to determine whether there were significant differences among the experimental treatment groups. When significant differences were detected (*p* < 0.05), Duncan's multiple range test was used for post hoc comparison of group means. All bar charts in this chapter are drawn using GraphPad Prism 6.0.

## 3. Results

### 3.1. Growth Performance and Feed Utilization

No significant difference in survival was observed among the different dietary treatment groups (*p* > 0.05, Table 3). However, abalones fed diets containing FO, PO, SO, and OO exhibited significantly higher FSL, FW, DISL, WGR, and SGR compared to those fed the LO diet (*p* < 0.05), with the FO diet showing the greatest improvement, followed by PO and OO diets. Furthermore, the FCR was significantly lower in the FO, PO, SO, and OO groups than in the LO group (*p* < 0.05).

### 3.2. Fertility

**Figure 1:**
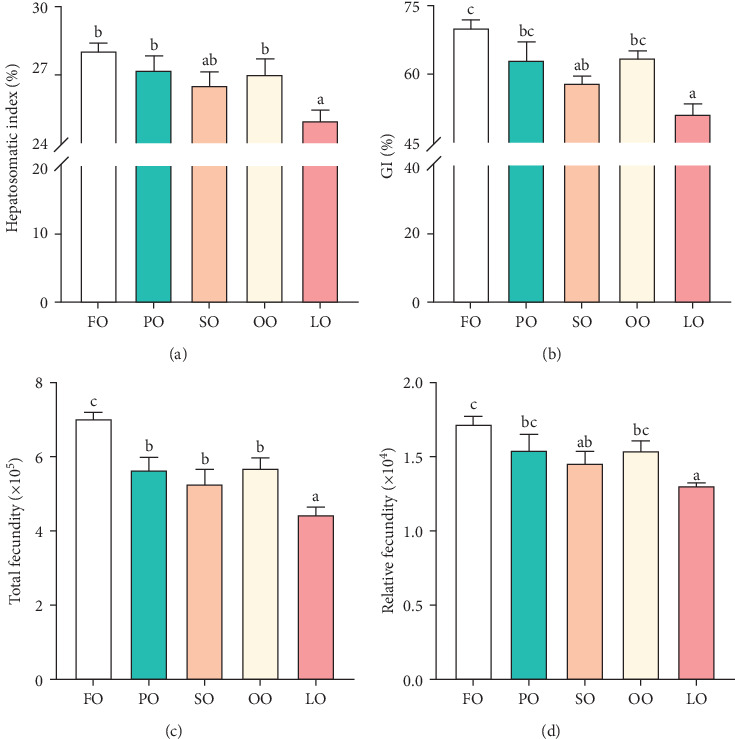
Parameters involved in fertility of female broodstock *Haliotis discus hannai* fed five experiment diets. (A) Hepatosomatic index (HSI), (B) gonad index (GI), (C) total fecundity, and (D) relative fecundity. Value columns with different letters mean significant differences (*p* < 0.05). FO, fish oil (from salmon oil); LO, lard; OO, olive oil; PO, perilla seed oil; SO, safflower oil.

As shown in Figure 1, the HSI, GI, total fecundity, and relative fecundity in the FO, PO, and OO groups were significantly higher than those in the LO group, with the highest values observed in the FO group (*p* < 0.05,). It should be noted that no significant differences were found in GI and relative fecundity among the PO, OO, and FO groups (*p* > 0.05, Figure 1B,D). Additionally, total fecundity in the FO group was significantly greater than that in both the vegetable oil groups and the LO group (*p* < 0.05, Figure 1C).

### 3.3. Reproductive Performance

**Figure 2:**
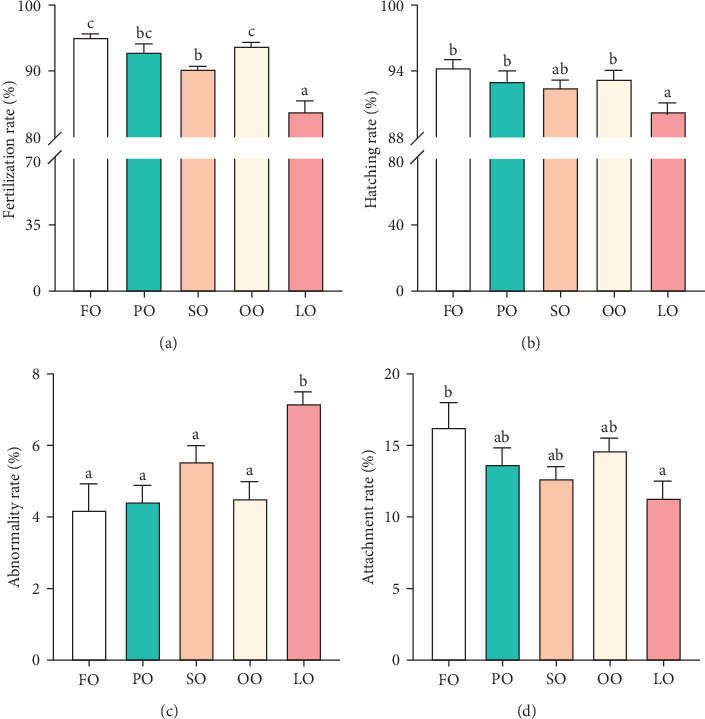
The reproductive performance of female broodstock *Haliotis discus hannai* fed five experiment diets. (A) Fertilization rate, (B) hatching rate, (C) abnormality rate, and (D) attachment rate. Value columns with different letters mean significant differences (p < 0.05). FO, fish oil (from salmon oil); LO, lard; OO, olive oil; PO, perilla seed oil; SO, safflower oil.

Figure 2 illustrates the effects of different dietary lipid sources on fertilization, hatching, abnormality, and attachment rates. The fertilization rate was significantly higher in the FO and PO groups compared to the LO group, with the highest rate observed in the FO group (*p* < 0.05, Figure 2A). Similarly, the hatching rate was significantly higher in the FO group than in the LO group, but no significant differences were found among the vegetable oil groups (*p* < 0.05, Figure 2B). The abnormality rate was significantly lower in the FO and vegetable oil groups compared to that in the LO group (*p* < 0.05, Figure 2C). Regarding the attachment rate, the FO group exhibited a significantly higher rate compared to that in the LO group (*p*<0.05, Figure 2D), but no significant differences were observed between the vegetable oil groups and the FO group (*p* > 0.05).

### 3.4. Histological Analysis

**Figure 3:**
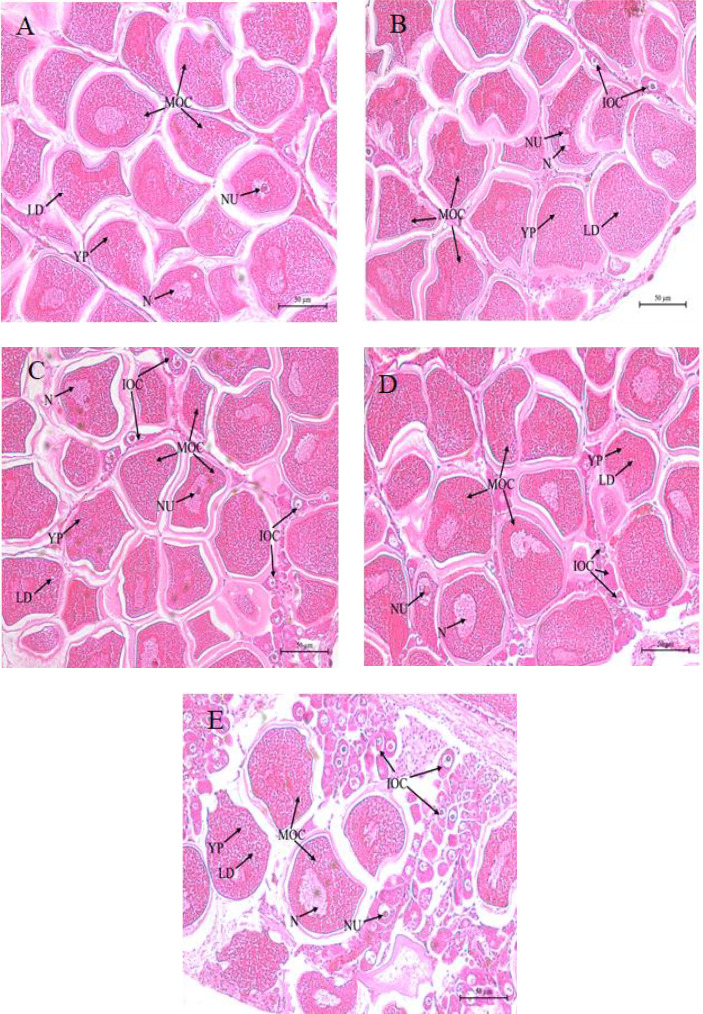
Histological images of the ovaries of female broodstock *Haliotis discus hannai* fed five experiment diets. (A) Fish oil, (B) perilla oil, (C) safflower oil, (D) olive oil, (E) lard. IOC: immature oocyte, LD: lipid droplet, MOC: mature oocyte, N: nucleus, NU: nucleolus, YP: yolk granule. Magnification 400×, scale bar 50 µm.

Histological analysis of the ovaries is shown in Figure 3. The FO group displayed a marked abundance of mature oocytes (Figure 3A), in contrast to the lower prevalence in the LO group (Figure 3E). The PO, SO, and OO groups predominantly exhibited mature oocytes, with only a few immature ones (Figure 3B–D). Notably, the FO and PO groups showed a greater accumulation of yolk granules and lipid droplets compared to those in the LO group.

### 3.5. Determination of the Biochemical and Hormone Parameters

**Figure 4:**
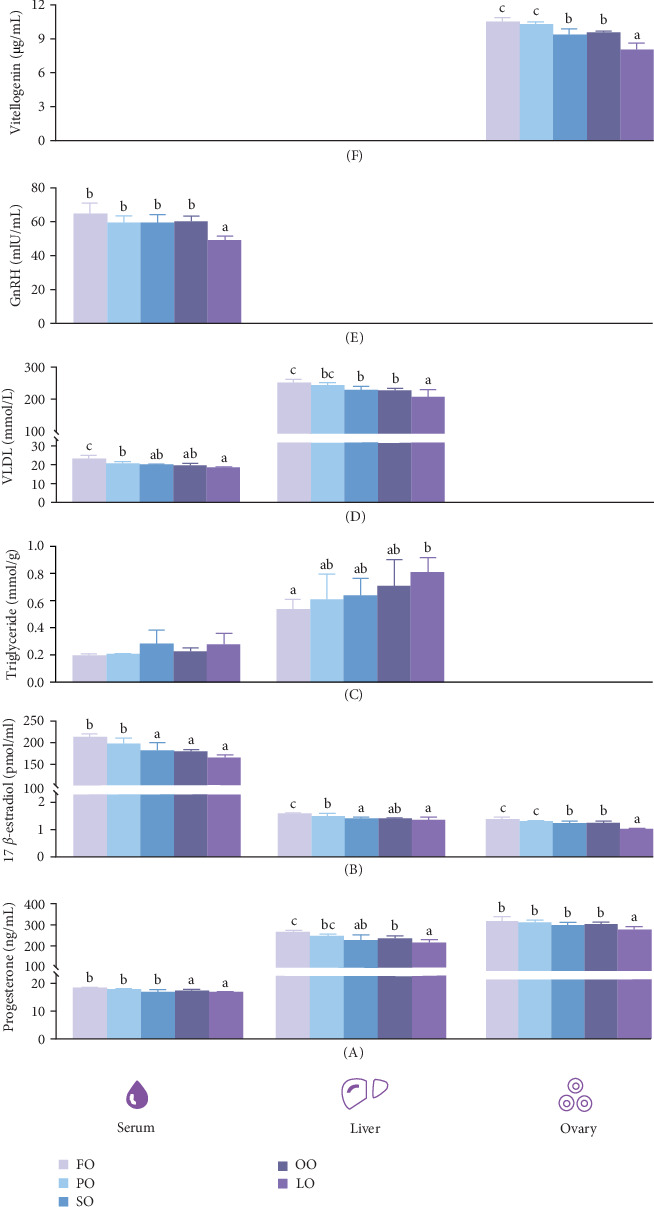
The levels of biochemical parameters and hormones in the serum, liver, and ovaries of female broodstock *Haliotis discus hannai* were measured after being fed with five experimental diets. The parameters and hormones measured were (A) progesterone (PROG), (B) 17 β-estradiol (E_2_), (C) triglyceride (TG), (D) very low-density lipoprotein (VLDL), (E) gonadotropin-releasing hormone (GnRH), and (F) vitellogenin (VTG). Columns with different letters indicate significant differences (*p* < 0.05). FO, fish oil (from salmon oil); LO, lard; OO, olive oil; PO, perilla seed oil; SO, safflower oil.

Figure 4 shows that the dietary treatment group did not significantly affect serum TG levels (*p* > 0.05). However, serum concentrations of VLDL, GnRH, PROG, and E_2_ were significantly higher in the FO and PO groups were compared to those in the LO group, with the FO group exhibiting the highest values (*p* < 0.05). Additionally, serum PROG and E_2_ levels in the FO and PO groups significantly greater than those in the other groups (*p* < 0.05). The dietary lipid source also significantly influenced liver TG, VLDL, PROG, and E_2_ levels (*p* < 0.05), with the FO group showing lower TG and higher VLDL levels compared to the LO group (*p* < 0.05). Furthermore, PROG and E_2_ levels in the FO group were also higher than those in the other groups, except for the PO group (*p* < 0.05). Ovarian VTG, PROG, and E_2_ levels were significantly influenced by the lipid source, with higher values in the FO and PO groups compared to those in the LO group (*p* < 0.05).

### 3.6. Fatty Acid Content

**Figure 5:**
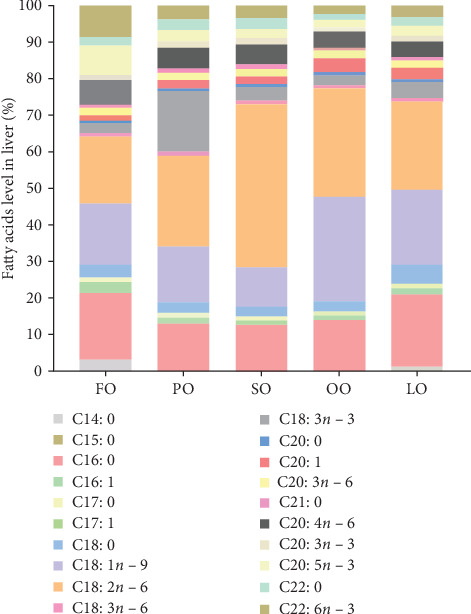
Stacked bar of the fatty acid composition (% total fatty acids) in the liver of female broodstock *Haliotis discus hannai* fed five lipid source diets. FO, fish oil (from salmon oil); LO, lard; OO, olive oil; PO, perilla seed oil; SO, safflower oil.

**Figure 6:**
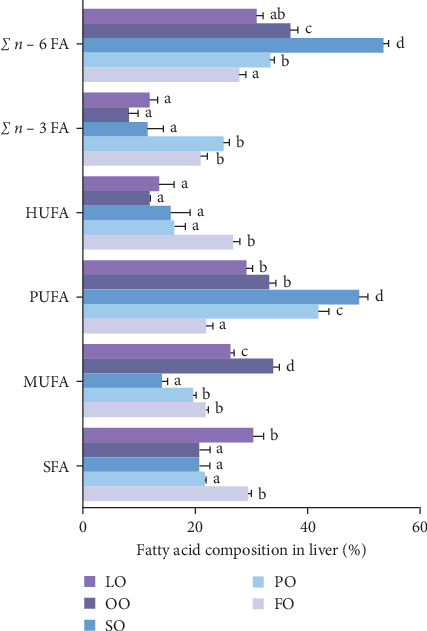
Effects of five dietary lipid sources on the fatty acid composition (% total fatty acids) in the ovary of female broodstock *Haliotis discus hannai*. SFA, saturated fatty acid; MUFA, monounsaturated fatty acid; PUFA, polyunsaturated fatty acids; HUFA, highly unsaturated fatty acids; *n −* 3 FAs, *n* − 3 FAs; *n*− 6 FAs, and *n −* 6 FAs. Means with different superscripts are significantly different (*p* < 0.05). FO, fish oil (from salmon oil); LO, lard; OO, olive oil; PO, perilla seed oil; SO, safflower oil.

**Figure 7:**
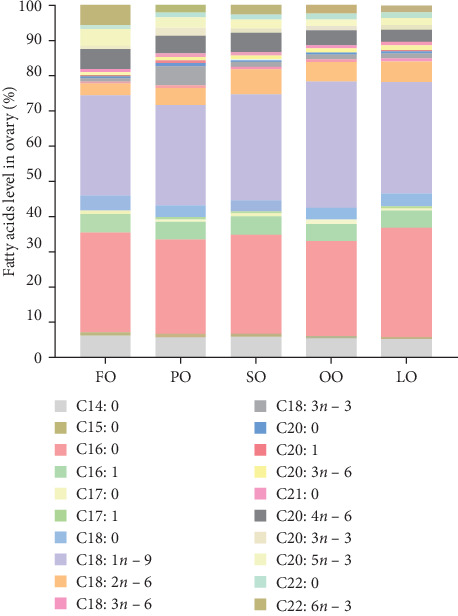
Stacked bar of the fatty acid composition (% total fatty acids) in the ovary of female broodstock *Haliotis discus hannai* fed five lipid source diets. FO, fish oil (from salmon oil); LO, lard; OO, olive oil; PO, perilla seed oil; SO, safflower oil; OO.

**Figure 8:**
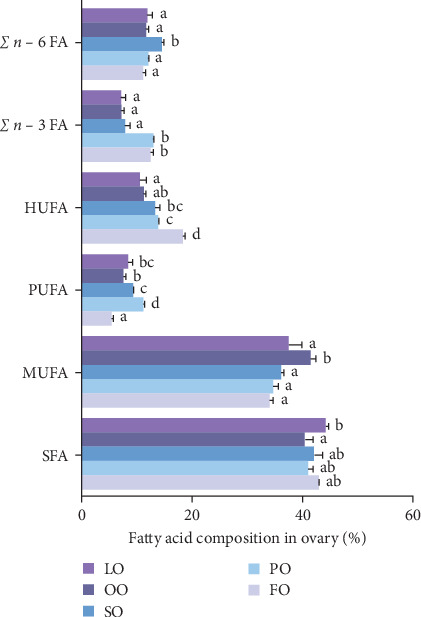
Effects of five dietary lipid sources on the fatty acid composition (% total fatty acids) in the liver of female broodstock *Haliotis discus hannai*. HUFA, highly unsaturated fatty acids; monounsaturated fatty acid; PUFA, polyunsaturated fatty acids;SFA, saturated fatty acid; *n −* 3 FA, *n −* 3 FAs; *n −* 6 FA, *n −* 6 FAs. Means with different superscripts are significantly different (*p* < 0.05). FO, fish oil (from salmon oil); LO, lard; OO, olive oil; PO, perilla seed oil; SO, safflower oil.

The fatty acid compositions in the liver and ovary are shown in Tables S2 and S3. Moreover, Figures 5–8 illustrated the fatty acid composition in the liver and ovary of female broodstock abalone across different dietary lipid treatments, respectively, corresponding to the data presented in supporting Table. The results show that dietary lipid sources significantly influence the fatty acid profile. In the livers, the LO group had significantly higher levels of SFA compared to the FO and vegetable oil groups (*p* < 0.05). The OO group had the highest levels of MUFA, particularly oleic acid (C18:1*n −* 9) (*p* < 0.05). The FO group resulted in the highest levels of LC-PUFAs, including EPA (C20:5*n −* 3) and DHA (C22:6*n −* 3) (*p* < 0.05). The SO group exhibited significantly higher LA (C18:2*n −* 6) levels, while the PO group showed higher ALA (C18:3*n −* 3) level (*p* < 0.05). In the ovaries, the FO group had notably higher levels of LC-PUFAs, especially EPA and DHA (*p* < 0.05). The PO group had significantly higher levels of PUFAs and C18:3*n −* 3, while the OO group had the highest level of C18:1*n −* 9, the LO group still exhibited the highest SFA levels (*p* < 0.05).

Among fatty acid compositions in the liver, the PO group had the highest levels of *n −* 3 (25.18% ± 0.90%) and *n −* 6 (33.35% ± 0.7%) fatty acids, followed by the FO group, with *n −* 3 and *n −* 6 fatty acids at 20.93% ± 1.24% and 27.80% ± 1.19%, respectively. The FO and PO groups were significantly higher than those SO and OO groups in the *n −* 3/*n −* 6 ratio (*p* < 0.05). In the ovaries, the FO and PO groups had the highest levels of *n* − 3 fatty acids (12.34% ± 0.36% and 12.69% ± 0.16%, respectively), while the level of *n −* 6 fatty acids in the SO group (14.41% ± 0.25%) was the highest (*p* < 0.05). And the levels of the *n −* 3/*n −* 6 ratio of the FO and PO groups were significantly higher than those of the other groups (*p* < 0.05).

## 4. Discussion

This study examined the effects of FO, PO, SO, OO, and LO on the growth and reproductive performance of female broodstock abalone (*H. discus hannai*). The fatty acid composition of these lipid sources varied significantly, influencing parameters, such as growth performance and biochemical composition. Like many marine species, abalone depend on LC-PUFAs such as EPA and DHA, and although abalone have the ability to biosynthesize LC-PUFAs from PUFAs, the efficiency of synthesis is influenced by the type and amount of dietary lipids [9, 24]. These EFAs are widely recognized for their role in enhancing growth performance [16]. While no significant differences in survival were observed among the dietary groups, lipid sources significantly influenced growth performance, consistent with previous studies [25]. FO and PO were particularly effective in promoting growth, likely due to their high levels of EFAs, FO provided abundant EPA and DHA, while PO contained LNA, which also supports growth [7, 26]. As previously reported, krill oil (enriched with LC-PUFAs) promotes the growth of swimming crab *P. trituberculatus* [8], and spirulina meal (enriched with LNA) has a positive effect on the growth performance of abalone (*H. iris*) [27]. Interestingly, the OO diet also improved growth performance and outperformed the LO diet. This may be attributed to the high MUFA content of OO, particularly oleic acid (C18:1*n −* 9), which serves as an efficient energy source, thereby sparing energy for growth [26]. The WGR and SGR in the FO group were comparable to those in the PO and OO groups, but significantly higher than those in the SO group. This suggests that, while LA in SO, is essential, its contribution to growth may be less significant than that of other fatty acids. Similar findings were reported in both abalone and tilapia, where growth rates were comparable in FO fed diets and those containing rapeseed or linseed oil [12, 28]. Conversely, the LO group exhibited a significantly higher FCR compared to other groups, which may be attributed to the absence of LC-PUFAs. Lard, being rich in SFAs, may offer lower digestibility and bioavailability for abalone, thereby necessitating greater feed intake to fulfill their nutritional requirements [12]. These findings highlight the critical role of dietary fatty acid composition in nutrient utilization and emphasize the importance of selecting lipid sources with a balanced profile of EFAs to optimize growth performance and feed efficiency in abalone.

Reproductive performance in aquatic species is strongly influenced by dietary nutrition, particularly the fatty acid composition of broodstock diets. LC-PUFAs are essential for promoting gonadal development and reproductive success [3, 29]. In this study, dietary lipid sources had a significant impact on gonadal development, fecundity, and related reproductive parameters, underscoring their critical role in broodstock nutrition for abalone. Among the treatment diets, the FO group exhibited the highest fecundity, which can be attributed to its high levels of EFAs, including EPA, DHA, and ALA. These fatty acids are known to support ovarian development and reproductive performance across various aquatic species, including *Channa striatus*, *Acipenser sinensis*, and *Eriocheir sinensis* [30–32]. The superior reproductive outcomes observed in the FO group reinforce the importance of providing adequate levels of LC-PUFAs in broodstock diets. However, partial or full replacement of FO with vegetable oils, such as OO, can alter lipid deposition in reproductive tissues and negatively affect reproductive outcomes [33]. Excessive substitution of FO with OO has been associated with undesirable lipid accumulation and reduced antioxidant capacity in *Larimichthys crocea* [34], as well as impaired ovarian development, decreased GI, and reductions in ovarian TG and LC-PUFAs levels in *Oreochromis niloticus* [29]. Similarly, *Strongylocentrotus intermedius* fed safflower seed oil exhibited lower GI compared to those fed FO [35]. In line with these findings, the OO group in this study exhibited lower GI, total fecundity, and relative fecundity, suggesting that, while the fatty acid profile of OO may be beneficial for somatic growth, it may not adequately support reproductive development. The SO group showed poor reproductive performance, likely due to its high LA content and lack of *n* - 3 LC-PUFAs [36]. Elevated dietary LA levels may impair steroid hormone synthesis, thereby delaying gonadal maturation, as reported in *C. carpio* [18] and *P. fulvidraco* [37]. In this study, the high LA content in the SO diet likely contributed to reduced fecundity and delayed gonadal development. The LO group exhibited the poorest reproductive performance, which can be attributed to its high SFA level. Saturated fats are less effective in supporting reproductive development, as observed in *Monopterus albus* fed lard-based diets [38]. Moreover, high dietary crude glycerol levels, often associated with lard-based feeds, have been shown to reduce reproductive performance in *O*. *niloticus*, resulting in lower egg production and hatching rates [39]. The poor reproductive outcomes observed in the LO group further confirm that diets deficient in LC-PUFAs and dominated by SFAs are suboptimal for supporting reproductive success in female broodstock abalone.

Reproductive performance in aquatic species is influenced by multiple factors, including GI, fertility, fertilization rates, hatching success, abnormality rates, and larval survival. These parameters collectively determine the reproductive efficiency of broodstock and the viability of their offspring. Nutritional components, particularly lipids, are critical in these processes [9]. Yolk reserves, which provide essential energy for larval attachment and metamorphosis, are highly dependent on the nutritional quality of broodstock diet [40]. A proper balance of LC-PUFAs is essential for successful gonad maturation, egg quality, and larval development [41]. Previous studies have demonstrated that supplementing broodstock diet with PUFAs significantly improves reproductive parameters, including fecundity, gamete viability, fertilization rates, hatching success, and larval survival in *Mystus cavasius*, dietary PUFAs were found to enhance gonad maturation and reproductive efficiency [9]. These findings highlight the critical role of PUFAs in supporting reproductive performance in aquatic species. In the present study, female broodstock abalone fed a diet with FO supplementation exhibited higher larval attachment rates and fewer developmental abnormalities, likely due to the high levels of *n −* 3 LC-PUFAs and other PUFAs in the diet. These EFAs play a vital role in supporting gonad development, improving egg quality, and ensuring healthier larval growth.

Traditional histological analysis provides critical insights into ovarian development by evaluating the size, development, and density of abalone oocytes [23]. Previous studies, such as those conducted on *C. striatus*, have reported that broodstock fed lipid-rich diets exhibited larger oocyte diameters, likely due to the accumulation of LC-PUFAs, which act as critical energy sources and precursors for eicosanoids [30]. In abalone, ovarian development is typically categorized into early maturing, late maturing, and ripe stages, based on gonad morphology and oocyte histology [42]. In the present study, histological examination revealed that abalone in the FO group reached the ripe stage, characterized by fully developed oocytes primed for spawning. In contrast, broodstock in the PO, SO, and OO groups showed large vitellogenic oocytes, indicating ovaries in a mature but not fully ripe state. Notably, abalone in the LO group exhibited immature ovaries containing round, irregular, and transparent oocytes lacking yolk, indicative of underdeveloped reproductive capacity. These observations highlight the pivotal role of dietary lipid sources, particularly the type and proportion of LC-PUFAs, in regulating ovarian development and oocyte maturation in female broodstock abalone. EFAs derived from dietary lipids are emulsified and transported from the hepatopancreas to the ovaries and other tissues, where they are utilized for lipid storage and energy supply during gametogenesis [3]. Similar nutrient transport and allocation mechanisms have been observed in other aquatic species, including *Litopenaeus vannamei* [43] and *P. trituberculatus* [44]. VLDL plays a central role in mediating lipid transport from the liver to peripheral tissues, and their assembly is influenced by the availability of key substrates such as TGs, phospholipids, and cholesterol [45]. In this study, FO and PO rich in LC-PUFAs and PUFAs, respectively, appeared to enhance VLDL assembly and secretion, potentially facilitating lipid mobilization, and reducing hepatic lipid accumulation. This observation is consistent with previous findings in *T. ovatus* [17], *Salmo salar* [24], and *P. trituberculatus* [46], where FO or linseed oil-based diets resulted in lower SFA deposition in the liver. Collectively, these results suggest that the fatty acid profile of the diet exerts both direct and indirect effects on lipid deposition and ovarian development in female broodstock abalone.

VTG is a well-established biomarker of reproductive status in oviparous animals and plays a fundamental role in ovarian development and maturation [47]. Its synthesis is tightly regulated by both endogenous and exogenous estrogens, which promote vitellogenesis by stimulating metabolic pathways such as lipogenesis and ion transport [48]. As a high-density glycoprotein composed of both lipids and proteins, VTG directly contributes to ovarian maturation by supplying essential nutrients for yolk formation [49]. Certain dietary fatty acids serve as important substrates for the synthesis of LC-PUFAs, which are crucial for VTG production. For example, LNA in perilla oil and LA in SO can be converted into EPA and ARA in *H. discus hannai* [50]. In the present study, broodstock abalone fed FO, PO, or SO diets exhibited elevated ovarian VTG levels, likely attributable to the availability of LC-PUFA precursors provided by these dietary lipid sources. Previous studies have demonstrated that diets enriched with EPA and DHA are associated with increased VTG concentrations, underscoring the importance of *n −* 3 PUFAs in supporting yolk protein synthesis and ovarian function [51]. Consistent with these findings, the FO and PO groups in this study showed significantly increased ovarian VTG levels, further supporting the beneficial role of *n −* 3 PUFAs in promoting vitellogenesis. These results emphasize the critical link between dietary lipid composition, LC-PUFA availability, and ovarian development in broodstock abalone.

Studies on *H*. *asinina* and *H*. *laevigata* have shown that GnRH is released into hemolymph vessels connected to the ganglion, where it is transported to the ovaries to stimulate steroidogenesis in estrogen-producing cells [52]. PUFAs play a pivotal role in this regulatory pathway by serving as precursors for bioactive signaling molecules such as eicosanoids, which modulate membrane signal transduction and influence steroid hormone activity. In addition, PUFAs can regulate the binding of steroid hormones, such as estrogen and PROG, to their intracellular receptors, thereby influencing steroid hormone feedback control [53]. Through these actions, PUFAs contribute significantly to the synthesis and regulation of steroid hormones, ultimately affecting ovarian development and reproductive function. PUFAs have also been reported to enhance hepatic steroid hormone synthesis and facilitate their accumulation in ovarian tissues [54]. In the present study, the FO diet resulted in higher PROG and *E*_2_ levels compared to the SO diet. This observation suggests that FO, which is rich in *n −* 3 LC-PUFAs such as EPA and DHA, effectively promotes the synthesis and secretion of key steroid hormones in abalone. Supporting evidence from studies in silver pompano indicates that dietary *n −* 3 LC-PUFAs not only elevate serum levels of follicle-stimulating hormone, luteinizing hormone, and *E*_2_ but also upregulate the expression of ovarian genes such as *cyp19α1 α*, which is involved in estradiol biosynthesis [55]. Furthermore, elevated levels of *n −* 3 LC-PUFAs have been associated with enhanced peroxisome proliferation and increased expression of genes involved in *E*_2_ synthesis [51]. These mechanistic insights help explain the elevated steroid hormone concentrations observed in the FO group compared to the SO group in this study. The presence of EPA and DHA in FO likely enhances GnRH stimulation and promotes the biosynthesis of PROG and *E*_2_, thereby facilitating VTG production and its accumulation in ovarian tissues. Collectively, these effects contribute to improved ovarian development and maturation, underscoring the critical role of dietary *n −* 3 LC-PUFAs in enhancing reproductive performance in female broodstock abalone.

## 5. Conclusion

This study investigated the effects of five dietary lipid sources on the growth, reproductive performance, biochemical composition, and hormone levels of female broodstock abalone *H. discus hannai*. Among the tested lipids, FO, which is rich in LC-PUFAs, was the most effective in enhancing overall broodstock performance. FO supplementation promoted the synthesis of GnRH and key steroid hormones, increased tissue LC-PUFA accumulation, and elevated ovarian VTG levels, collectively supporting improved ovarian maturation and reproductive efficiency. Despite its benefits, the limited supply and high cost of FO necessitate the search for alternatives. PO, rich in EFA substrates like LNA, showed potential as a viable substitute, supporting growth, and reproductive health. These findings highlight the crucial role of dietary lipid composition in optimizing broodstock performance. While FO remains the most effective lipid source, vegetable-based alternatives such as PO offer a sustainable and cost-effective option for improving reproductive outcomes in abalone aquaculture.

## Figures and Tables

**Table 1:** Ingredient formulation (dry matter basis, g/kg) and proximate composition (dry matter basis, %) of the experimental diets.

Ingredients	Experiment diets				
	FO	PO	SO	OO	LO
Fish meal^a^	20	20	20	20	20
Wheat gluten^a^	90	90	90	90	90
Soybean meal^a^	265	265	265	265	265
High gluten flour^a^	250	250	250	250	250
Kelp powder^a^	300	300	300	300	300
Fish oil (FO)^b^	20	—	—	—	—
Perilla oil (PO)^c^	—	20	—	—	—
Safflower oil (SO)^d^	—	—	20	—	—
Olive oil (OO)^e^	—	—	—	20	—
Lard (LO)^f^	—	—	—	—	20
Soy lecithin^g^	5	5	5	5	5
Cholesterol^h^	3	3	3	3	3
Monocalcium phosphate	20	20	20	20	20
Vitamin premix^i^	10	10	10	10	10
Mineral premix^j^	10	10	10	10	10
Choline chloride	5	5	5	5	5
Ethoxyquin	1	1	1	1	1
Calcium propionate	1	1	1	1	1
Proximate composition					
Moisture	4.39 ± 0.05	4.40 ± 0.38	4.50 ± 0.13	4.29 ± 0.13	4.47 ± 0.20
Crude protein	36.37 ± 0.37	36.30 ± 0.69	36.56 ± 0.38	36.83 ± 0.96	36.16 ± 0.62
Total lipid	4.04 ± 0.17	4.01 ± 0.13	4.10 ± 0.48	4.12 ± 0.30	4.16 ± 0.39
Ash	17.77 ± 0.24	17.51 ± 0.60	17.81 ± 1.77	17.79 ± 1.00	17.10 ± 0.89

^a^Provided by Fuzhou Bohai Biotechnology Co. Ltd. Fuzhou, China. Fish meal: anchovy fishmeal imported from Peru, 67.19% of crude protein, 10.84% of crude lipid.

^b^The salmon (*Oncorhynchus* spp.) extract, obtained from Fujian Gaolong Marine Bioengineering Co. Ltd. (Fujian, China), contained 5.99% EPA and DHA.

^c^Purchased from Jilin Changbai Workshop Technology and Trade Co. Ltd. (Jilin, China), contained 64.3% ALA.

^d^Purchased from Zhejiang Jiusheng Camellia Technology Co. Ltd. (Zhejiang, China), contained 67.8% LA.

^e^Purchased from Shanghai Sinuosuola Trading Co. Ltd. (Shanghai, China), contained 79.0% MUFA.

^f^Purchased from Henan Luohe Shuanghui Edible Oil Technology Co. Ltd. (Henan, China), contained 43.2% SFA.

^g^Purchased from Shanghai Taiwei Pharmaceutical Co. Ltd. Shanghai, China, contained phosphatidylcholine 55.9%, phosphatidylethanolamine: 19.4%, phosphatidylinositol 10%.

^h^Purchased from Shanghai Maclean Biochemical Technology Co. Ltd. Shanghai, China, Cholesterol purity 99.0%

^i^Vitamin complex (per kg): vitamin A, 2.01 g; vitamin D3, 0.41 g; vitamin E, 0.02 g; vitamin K3, 3.4 g; vitamin B1, 6.7 g; vitamin B2, 10 g; vitamin B6, 8 g; vitamin B12, 0.01; vitamin C, 100 g; folic acid, 3.3 g; D-pantothenic acid; 26.5 g; niacinamide, 53.5 g; inositol, 80 g; D-biotin, 0.34 g; fill with skimmed rice bran to 1000 g.

^j^Mineral complex (per kg): copper sulfate, 1 g; ferrous sulfate, 12 g; manganese sulfate, 1 g; magnesium sulfate, 8 g; zinc sulfate, 5 g; potassium iodate, 0.065 g; sodium selenite, 0.025 g; cobalt chloride, 0.06 g; defatted rice bran, 200 g; fill with zeolite powder to 1000 g.

**Table 2:** Fatty acid composition (% total fatty acids) of experiment diets.

Fatty acids	Experiment diets				
	FO	PO	SO	OO	LO
C14:0	1.29	0.00	0.00	0.00	0.58
C15:0	0.24	0.02	0.07	0.08	0.10
C16:0	23.43	11.67	12.77	10.76	34.89
C16:1	2.45	0.72	0.75	1.18	1.93
C17:0	0.05	0.00	0.00	0.00	0.06
C17:1	0.08	0.05	0.06	0.11	0.09
C18:0	3.44	3.20	3.18	4.12	20.12
C18:1*n −* 9	18.79	15.80	12.38	49.83	23.65
C18:2*n −* 6	20.59	14.91	56.35	19.79	4.40
C18:3*n −* 6	1.75	0.85	0.97	0.91	0.83
C18:3*n −* 3	3.49	43.59	3.06	2.39	3.01
C20:0	0.83	0.54	0.69	0.72	0.63
C20:1	1.35	0.59	0.69	1.25	0.86
C20:3*n −* 6	0.89	0.80	0.94	0.90	0.92
C21:0	0.52	0.45	0.52	0.50	0.47
C20:4*n −* 6	2.79	1.52	1.65	1.77	1.86
C20:3*n −* 3	2.06	0.89	1.03	0.95	0.94
C20:5*n −* 3	4.92	1.43	1.53	1.54	1.62
C22:0	2.60	1.19	1.39	1.31	1.24
C22:6*n −* 3	8.44	1.76	1.98	1.90	1.81
SFA^a^	31.88	16.63	18.09	16.98	57.61
MUFA^b^	22.67	17.17	13.88	52.37	26.53
PUFA^c^	25.83	59.34	60.38	23.08	8.23
HUFA^d^	19.10	6.40	7.13	7.06	7.16
*Σ n −* 3 FA^e^	18.91	47.67	7.60	6.78	7.38
*Σ n −* 6 FA^f^	26.02	18.07	59.92	23.37	8.01
*Σ n −* 3 FA/*Σ n −* 6 FA	0.73	2.64	0.13	0.29	0.92

Abbreviations: FO, fish oil (from salmon oil); LO, lard; OO, olive oil; PO, perilla seed oil; SO, safflower oil.

^a^SFA (saturated fatty acid): C14:0, C15:0, C16:0, C17:0, C18:0, C20:0, C21:0, C22:0.

^b^MUFA (monounsaturated fatty acid): C16:1, C17:1, C18:1*n −* 9, C20:1.

^c^PUFA (polyunsaturated fatty acid): C18:2*n −* 6, C18:3*n −* 6, C18:3*n −* 3.

^d^HUFA (highly unsaturated fatty acid): C20:3*n −* 6, C20:4*n −* 6, C20:3*n −* 3, C20:5*n −* 3, C22:6*n −* 3.

^e^ *n −* 3 FA (*n −* 3 fatty acid): C18:3*n −* 3, C20:3*n −* 3, C20:5*n −* 3, C22:6*n −* 3.

^f^ *n −* 6 FA (*n −* 6 fatty acid): C18:2*n −* 6, C18:3*n* − 6, C20:3*n −* 6, C20:4*n −* 6.

**Table 3:** Growth performance of female broodstock *Haliotis discus hannai*.

Parameters	Experiment diets				
	FO	PO	SO	OO	LO
Survival (%)	99.75 ± 0.25^a^	99.00 ± 0.41^a^	98.75 ± 0.63^a^	99.25 ± 0.25^a^	99.25 ± 0.48^a^
FSL (mm)	68.76 ± 0.47^b^	68.38 ± 0.45^b^	67.97 ± 0.63^b^	68.06 ± 0.73^b^	65.99 ± 0.47^a^
FW (g)	46.36 ± 0.88^c^	44.51 ± 0.92^b,c^	42.53 ± 0.69^b^	44.43 ± 1.23^b,c^	38.69 ± 1.23^a^
DISL (µm/day)	84.27 ± 4.74^b^	80.51 ± 4.54^b^	76.40 ± 6.30^b^	77.30 ± 7.31^b^	56.60 ± 4.74^a^
WGR (%)	64.58 ± 3.13^c^	57.99 ± 3.26^b,c^	50.98 ± 2.46^b^	57.72 ± 4.35^b,c^	37.34 ± 4.35^a^
SGR (%)	0.55 ± 0.02^c^	0.51 ± 0.02^b,c^	0.46 ± 0.02^b^	0.50 ± 0.03^b,c^	0.35 ± 0.03^a^
FCR	1.89 ± 0.13^a^	1.99 ± 0.12^a^	2.12 ± 0.09^a^	2.03 ± 0.17^a^	2.80 ± 0.34^b^

Note: The data were expressed as mean ± standard error. Values in the same line with different superscript letters are significantly different (*p* < 0.05).

Abbreviations: DISL, daily increment in shell length; FCR, feed conversion rate; FO, fish oil (from salmon oil); FSL, final shell length; FW, final body weight; LO, lard; OO, olive oil; PO, perilla seed oil; SGR, specific growth rate; SO, safflower oil; WGR, weight gain rate.

## Data Availability

The data supporting this study's findings are available from the corresponding author upon reasonable request.

## Nomenclature

HUFA: Highly unsaturated fatty acid
LC-PUFA: Long-chain polyunsaturated fatty acid
PUFA: Polyunsaturated fatty acid
SFA: Saturated fatty acid
MUFA: Monounsaturated fatty acid
*n −* 3 FA: *n −* 3 fatty acid
*n −* 6 FA: *n −* 6 fatty acid
LA: Linoleic acid
LNA: Linolenic acid
ARA: Arachidonic acid
EPA: Eicosapentaenoic acid
DHA: Docosahexaenoic acid
VLDL: Very low-density lipoprotein
GI: Gonad index
GnRH: Gonadotropin-releasing hormone
PROG: Progesterone
E_2_: 17 β-estradiol
VTG: Vitellogenin.

## Supporting Information

Supporting Information Table S1: The retention efficiency of the experimental diets at different immersion times in seawater. Table S2: Effects of dietary different lipid sources on the fatty acid composition (% total fatty acids) in the liver of female broodstock *Haliotis discus hannai*. Table S3: Effects of dietary different lipid sources on the fatty acid composition (% total fatty acids) in the ovary of female broodstock *H. discus hannai*.
