# Supplementary material for: Dietary Supplementation With Fish Oil Enhances the Growth and Reproductive Performance of Female Broodstock Haliotis discus hannai
Source: Aquac Nutr. 2025 Jul 15;2025:9987051. doi: 10.1155/anu/9987051 (PMC12283210; doi:10.1155/anu/9987051)
Supplement: Supporting Information — Table S1: The retention efficiency of the experimental diets at different immersion times in seawater. Table S2: Effects of dietary different lipid sources on the fatty acid composition (% total fatty acids) in the liver of female broodstock Haliotis discus hannai. Table S3: Effects of dietary different lipid sources on the fatty acid composition (% total fatty acids) in the ovary of female broodstock H. discus hannai. [file 9987051.f1.docx]

Supplementary Table 1. The retention efficiency of the experimental diets at different immersion times in seawater

| Parameters | Experiment diets | | | | |
| --- | --- | --- | --- | --- | --- |
|  | FO | PO | SO | OO | LO |
| Immersion for 24 h (%) | 85.25 ± 0.62 | 85.40 ± 0.92 | 85.21 ± 0.45 | 85.46 ± 0.16 | 85.49 ± 0.48 |
| Immersion for 48 h (%) | 82.33 ± 0.60 | 82.23 ± 0.42 | 82.16 ± 0.24 | 82.60 ± 0.36 | 82.32 ± 0.09 |

FO, fish oil (from salmon oil); PO, perilla seed oil; SO, safflower oil; OO, olive oil; LO, lard.

Supplementary Table 2. Effects of dietary different lipid sources on the fatty acid composition (% total fatty acids) in the liver of female broodstock *Haliotis discus hannai.*

| Fatty acids | Experiment diets | | | | |
| --- | --- | --- | --- | --- | --- |
|  | FO | PO | SO | OO | LO |
| C14:0 | 2.63 ± 0.13^b^ | 0.14 ± 0.14^a^ | 0.73 ± 0.58^a^ | 0.69 ± 0.64^a^ | 0.89 ± 0.89^ab^ |
| C15:0 | 0.60 ± 0.08 | 0.24 ± 0.08 | 0.33 ± 0.17 | 0.33 ± 0.10 | 0.47 ± 0.13 |
| C16:0 | 18.06 ± 0.55^bc^ | 12.70 ± 0.77^ab^ | 11.61 ± 2.71^a^ | 12.98 ± 1.71^ab^ | 19.33 ± 1.88^c^ |
| C16:1 | 3.12 ± 0.09^d^ | 1.46 ± 0.10^b^ | 1.08 ± 0.12^a^ | 1.19 ± 0.07^ab^ | 1.88 ± 0.10^c^ |
| C17:0 | 0.90 ± 0.03 | 0.80 ± 0.13 | 0.76 ± 0.21 | 0.62 ± 0.07 | 0.82 ± 0.12 |
| C17:1 | 0.29 ± 0.03 | 0.36 ± 0.10 | 0.32 ± 0.12 | 0.19 ± 0.05 | 0.27 ± 0.06 |
| C18:0 | 3.35 ± 0.05^b^ | 3.09 ± 0.04^ab^ | 2.84 ± 0.11^a^ | 3.24 ± 0.12^ab^ | 5.24 ± 0.22^c^ |
| C18:1n-9 | 16.92 ± 0.43^b^ | 15.5 ± 0.64^b^ | 10.71 ± 1.23^a^ | 28.51 ± 0.9^d^ | 20.87 ± 0.87^c^ |
| C18:2n-6 | 18.33 ± 1.27^a^ | 24.63 ± 1.36^b^ | 44.68 ± 1.96^d^ | 29.67 ± 0.75^c^ | 23.92 ± 1.26^b^ |
| C18:3n-6 | 0.90 ± 0.03 | 1.13 ± 0.23 | 1.08 ± 0.41 | 0.77 ± 0.18 | 1.02 ± 0.25 |
| C18:3n-3 | 2.72 ± 0.09^a^ | 16.40 ± 0.49^c^ | 3.53 ± 0.23^ab^ | 2.84 ± 0.31^a^ | 4.34 ± 0.52^b^ |
| C20:0 | 0.72 ± 0.02 | 0.83 ± 0.17 | 0.8 ± 0.31 | 0.55 ± 0.12 | 0.7 ± 0.18 |
| C20:1 | 1.52 ± 0.13^a^ | 2.38 ± 0.19^a^ | 2.04 ± 0.44^a^ | 4.05 ± 0.25^b^ | 3.28 ± 0.24^b^ |
| C20:3n-6 | 1.96 ± 0.19 | 1.99 ± 0.27 | 2.16 ± 0.56 | 2.02 ± 0.31 | 1.96 ± 0.40 |
| C21:0 | 0.91 ± 0.04 | 1.12 ± 0.19 | 1.08 ± 0.33 | 0.75 ± 0.15 | 0.99 ± 0.22 |
| C20:4n-6 | 6.62 ± 0.14^b^ | 5.60 ± 0.16^b^ | 5.54 ± 0.31^b^ | 4.42 ± 0.38^a^ | 4.10 ± 0.52^a^ |
| C20:3n-3 | 1.51 ± 0.03 | 1.90 ± 0.46 | 1.77 ± 0.75 | 1.16 ± 0.31 | 1.61 ± 0.49 |
| C20:5n-3 | 8.09 ± 0.59^b^ | 3.13 ± 0.31^a^ | 2.71 ± 0.70^a^ | 2.01 ± 0.36^a^ | 2.89 ± 0.53^a^ |
| C22:0 | 2.25 ± 0.04 | 2.84 ± 0.65 | 2.66 ± 1.15 | 1.72 ± 0.47 | 2.31 ± 0.70 |
| C22:6n-3 | 8.61 ± 0.59^b^ | 3.76 ± 0.63^a^ | 3.57 ± 1.18^a^ | 2.28 ± 0.58^a^ | 3.09 ± 0.73^a^ |
| SFA^1^ | 29.43 ± 0.6^b^ | 21.75 ± 0.24^a^ | 20.81 ± 1.74^a^ | 20.89 ± 1.79^a^ | 30.76 ± 1.90^b^ |
| MUFA^2^ | 21.84 ± 0.52^b^ | 19.71 ± 0.45^b^ | 14.15 ± 0.93^a^ | 33.95 ± 1.04^d^ | 26.30 ± 0.72^c^ |
| PUFA^3^ | 21.94 ± 1.29^a^ | 42.16 ± 1.62^c^ | 49.3 ± 1.40^d^ | 33.28 ± 1.12^b^ | 29.28 ± 0.97^b^ |
| HUFA^4^ | 26.79 ± 1.16^b^ | 16.38 ± 1.82^a^ | 15.75 ± 3.36^a^ | 11.88 ± 1.91^a^ | 13.65 ± 2.63^a^ |
| n-3 FA^5^ | 20.93 ± 1.24^b^ | 25.18 ± 0.90^b^ | 11.58 ± 2.78^a^ | 8.29 ± 1.52^a^ | 11.93 ± 1.43^a^ |
| n-6 FA^6^ | 27.80 ± 1.19^a^ | 33.35 ± 0.7^b^ | 53.47 ± 0.93^d^ | 36.88 ± 1.41^c^ | 31.00 ± 1.11^ab^ |
| n-3 FA/n-6 FA | 0.76 ± 0.07^b^ | 0.76 ± 0.04^b^ | 0.22 ± 0.06^a^ | 0.22 ± 0.04^a^ | 0.38 ± 0.04^a^ |

FO, fish oil (from salmon oil); PO, perilla seed oil; SO, safflower oil; OO, olive oil; LO, lard.

^1^ SFA (Saturated fatty acid): C14:0, C15:0, C16:0, C17:0, C18:0, C20:0, C21:0, C22:0.

^2^ MUFA (Monounsaturated fatty acid): C16:1, C17:1, C18:1n-9, C20:1.

^3^ PUFA (Polyunsaturated fatty acid): C18:2n-6, C18:3n-6, C18:3n-3.

^4^ HUFA (Highly unsaturated fatty acid): C20:3n-6, C20:4n-6, C20:3n-3, C20:5n-3, C22:6n-3.

^5^ n-3 FA (n-3 fatty acid): C18:3n-3, C20:3n-3, C20:5n-3, C22:6n-3.

^6^ n-6 FA (n-6 fatty acid): C18:2n-6, C18:3n-6, C20:3n-6, C20:4n-6.

Supplementary Table 3 Effects of dietary different lipid sources on the fatty acid composition (% total fatty acids) in the ovary of female broodstock *Haliotis discus hannai.*

| Fatty acids | Experiment diets | | | | |
| --- | --- | --- | --- | --- | --- |
|  | FO | PO | SO | OO | LO |
| C14:0 | 6.30 ± 0.63 | 5.83 ± 0.33 | 5.89 ± 0.63 | 5.47 ± 0.65 | 5.26 ± 1.19 |
| C15:0 | 0.72 ± 0.06 | 0.69 ± 0.02 | 0.72 ± 0.06 | 0.60 ± 0.05 | 0.56 ± 0.12 |
| C16:0 | 28.47 ± 0.26^ab^ | 27.09 ± 0.68^a^ | 28.11 ± 1.17^ab^ | 27.07 ± 1.28^a^ | 30.88 ± 0.93^b^ |
| C16:1 | 5.17 ± 0.08 | 4.76 ± 0.36 | 5.27 ± 0.07 | 4.81 ± 0.01 | 4.91 ± 0.34 |
| C17:0 | 0.76 ± 0.07 | 0.81 ± 0.01 | 0.9 ± 0.04 | 0.83 ± 0.06 | 0.81 ± 0.05 |
| C17:1 | 0.27 ± 0.10^a^ | 0.45 ± 0.03^ab^ | 0.44 ± 0.03^ab^ | 0.46 ± 0.02^b^ | 0.47 ± 0.05^b^ |
| C18:0 | 4.15 ± 0.16^c^ | 3.57 ± 0.04^ab^ | 3.26 ± 0.06^a^ | 3.26 ± 0.09^a^ | 3.63 ± 0.14^b^ |
| C18:1n-9 | 28.31 ± 0.43^a^ | 28.53 ± 0.74^a^ | 30.17 ± 0.57^a^ | 36.02 ± 0.89^b^ | 31.53 ± 1.77^a^ |
| C18:2n-6 | 3.65 ± 0.06^a^ | 4.81 ± 0.11^b^ | 6.93 ± 0.14^d^ | 5.31 ± 0.07^bc^ | 5.98 ± 0.48^c^ |
| C18:3n-6 | 0.54 ± 0.13 | 0.65 ± 0.02 | 0.79 ± 0.10 | 0.76 ± 0.14 | 0.77 ± 0.14 |
| C18:3n-3 | 0.93 ± 0.15^a^ | 5.46 ± 0.11^c^ | 1.37 ± 0.10^b^ | 1.33 ± 0.11^b^ | 1.47 ± 0.11^b^ |
| C20:0 | 0.6 ± 0.09 | 0.91 ± 0.23 | 0.56 ± 0.06 | 0.60 ± 0.14 | 0.53 ± 0.08 |
| C20:1 | 0.19 ± 0.19^a^ | 0.74 ± 0.12^b^ | 0.00 ± 0.00^a^ | 0.04 ± 0.04^a^ | 0.43 ± 0.21^ab^ |
| C20:3n-6 | 0.95 ± 0.00^a^ | 1.12 ± 0.02^b^ | 1.31 ± 0.02^c^ | 1.12 ± 0.05^b^ | 1.31 ± 0.05^c^ |
| C21:0 | 0.75 ± 0.12 | 0.83 ± 0.03 | 0.99 ± 0.13 | 0.95 ± 0.16 | 0.98 ± 0.19 |
| C20:4n-6 | 5.75 ± 0.16^c^ | 5.27 ± 0.08^c^ | 5.38 ± 0.25^c^ | 4.25 ± 0.18^b^ | 3.54 ± 0.30^a^ |
| C20:3n-3 | 0.86 ± 0.21^a^ | 1.99 ± 0.00^b^ | 1.25 ± 0.15^a^ | 1.17 ± 0.16^a^ | 1.23 ± 0.20^a^ |
| C20:5n-3 | 4.88 ± 0.04^c^ | 3.06 ± 0.05^b^ | 2.50 ± 0.15^a^ | 2.02 ± 0.23^a^ | 2.03 ± 0.24^a^ |
| C22:0 | 1.08 ± 0.26 | 1.25 ± 0.02 | 1.53 ± 0.19 | 1.45 ± 0.23 | 1.51 ± 0.29 |
| C22:6n-3 | 5.67 ± 0.47^b^ | 2.19 ± 0.04^a^ | 2.63 ± 0.30^a^ | 2.48 ± 0.37^a^ | 2.15 ± 0.38^a^ |
| SFA^1^ | 42.82 ± 0.05^ab^ | 40.97 ± 0.79^ab^ | 41.95 ± 1.47^ab^ | 40.23 ± 1.47^a^ | 44.17 ± 0.39^b^ |
| MUFA^2^ | 33.95 ± 0.49^a^ | 34.48 ± 0.94^a^ | 35.88 ± 0.56^a^ | 41.33 ± 0.89^b^ | 37.34 ± 2.27^a^ |
| PUFA^3^ | 5.12 ± 0.27^a^ | 10.92 ± 0.15^d^ | 9.1 ± 0.10^c^ | 7.41 ± 0.30^b^ | 8.23 ± 0.73^bc^ |
| HUFA^4^ | 18.11 ± 0.35^d^ | 13.63 ± 0.10^c^ | 13.06 ± 0.86^bc^ | 11.03 ± 0.34^ab^ | 10.27 ± 1.17^a^ |
| n-3 FA^5^ | 12.34 ± 0.36^b^ | 12.69 ± 0.16^b^ | 7.76 ± 0.70^a^ | 7.00 ± 0.41^a^ | 6.89 ± 0.93^a^ |
| n-6 FA^6^ | 10.89 ± 0.28^a^ | 11.86 ± 0.07^a^ | 14.41 ± 0.25^b^ | 11.44 ± 0.27^a^ | 11.61 ± 0.97^a^ |
| n-3 FA/n-6 FA | 1.14 ± 0.05^b^ | 1.07 ± 0.01^b^ | 0.54 ± 0.04^a^ | 0.61 ± 0.03^a^ | 0.59 ± 0.03^a^ |

FO, fish oil (from salmon oil); PO, perilla seed oil; SO, safflower oil; OO, olive oil; LO, lard.

^1^ SFA (Saturated fatty acid): C14:0, C15:0, C16:0, C17:0, C18:0, C20:0, C21:0, C22:0.

^2^ MUFA (Monounsaturated fatty acid): C16:1, C17:1, C18:1n-9, C20:1.

^3^ PUFA (Polyunsaturated fatty acid): C18:2n-6, C18:3n-6, C18:3n-3.

^4^ HUFA (Highly unsaturated fatty acid): C20:3n-6, C20:4n-6, C20:3n-3, C20:5n-3, C22:6n-3.

^5^ n-3 FA (n-3 fatty acid): C18:3n-3, C20:3n-3, C20:5n-3, C22:6n-3.

^6^ n-6 FA (n-6 fatty acid): C18:2n-6, C18:3n-6, C20:3n-6, C20:4n-6.
